# Quality of care for children with acute malnutrition at health center level in Uganda: a cross sectional study in West Nile region during the refugee crisis

**DOI:** 10.1186/s12913-018-3366-5

**Published:** 2018-07-17

**Authors:** Humphrey Wanzira, Richard Muyinda, Peter Lochoro, Giovanni Putoto, Giulia Segafredo, Henry Wamani, Marzia Lazzerini

**Affiliations:** 1 WHO Collaborating Centre for Maternal and Child Health, Institute for Maternal and Child Health IRCCS Burlo Garofolo, Trieste, Italy; 2 Doctors with Africa, CUAMM, Kampala, Uganda; 3 Makerere University School of Public Health, Kampala, Uganda

**Keywords:** Acute malnutrition, Children under 5 years, Quality of care, Quality assessment, Health center

## Abstract

**Background:**

Arua district, in Uganda, hosts some of the largest refugee camps in the country. The estimated prevalence of moderate and severe acute malnutrition in children is higher than the national estimates (10.4 and 5.6% respectively, compared to 3.6 and 1.3%). This study aimed at assessing the quality of care provided to children with acute malnutrition at out-patient level in such a setting.

**Methods:**

Six facilities with the highest number of children with malnutrition were selected. The main tool used was the National Nutrition Service Delivery Assessment Tool, assessing 10 key areas of service delivery and assigned a score as either poor, fair, good or excellent. Health outcomes, quality of case management and data quality were assessed from the health management information system and from the official nutrition registers.

**Results:**

All facilities except two scored either poor or fair under all the 10 assessment areas. Overall, 33/60 (55%) areas scored as poor, 25/60 (41%) as fair, 2/60 (3.3%) as good, and none as excellent. Main gaps identified included: lack of trained staff; disorganised patient flow; poor case management; stock out of essential supplies including ready-to-use therapeutic foods; weak community linkage. A sample coverage of 45.4% (1020/2248) of total children admitted in the district during the 2016 financial year were included. The overall mean cure rate was 52.9% while the default rate was 38.3%. There was great heterogeneity across health facilities in health outcomes, quality of case management, and data quality.

**Conclusion:**

This study suggests that quality of care provided to children with malnutrition at health center level is substandard with unacceptable low cure rates. It is essential to identify effective approaches to enhance adherence to national guidelines, provision of essential nutritional commodities, regular monitoring of services and better linkage with the community through village health teams.

**Electronic supplementary material:**

The online version of this article (10.1186/s12913-018-3366-5) contains supplementary material, which is available to authorized users.

## Background

Under-nutrition is a major cause of morbidity in children under 5 years [1]. The most recent estimates indicate that 52 million children under 5 years are diagnosed with wasting and 17 million with severe wasting and of these, 26.9% occur in Sub-Saharan Africa [1].

In Uganda, under-nutrition is considered a condition of public health importance [2]. National estimates report that 3.6% children suffer from moderate acute malnutrition (MAM) while 1.3% have severe acute malnutrition (SAM) [3]. However, this prevalence is heterogeneous across regions. For instance, the West Nile region, currently considered as a humanitarian setting and hosting refuges from South Sudan and Congo [4, 5] has the highest reported prevalence of MAM and SAM in the country at 10.4 and 5.6% respectively [3]. This is far above the target identified by the World Health Assembly which adopted the goal of reducing and maintaining the prevalence of wasting in children to under 5% by 2025 [6, 7].

Uganda is committed to reducing malnutrition and has identified this as a key part of its strategy for becoming a middle-income country by 2040 [8]. Actions to address malnutrition were included in the National Development Plan 2015/2016–2019/20 [9] and in the Uganda Nutrition Action Plan 2011–2016 for multi-sectoral support [10]. The Ministry of Health (MoH) developed the Integrated Management of Acute Malnutrition (IMAM) guidelines in 2006 and updated them in 2011 [11] and 2016 [2], in line with the WHO recommendations. The guidelines provide details for the management of children with both MAM and SAM at health facility level and include recommendations for screening and follow up at community level. Support has been provided from both international and local stakeholders especially the procurement and distribution of therapeutic foods and basic nutritional equipment [10, 12].

However, several studies have shown that adopting guidelines, providing training and basic equipment per se do not actually ensure that care is delivered according to set standards [13–17]. Assessments of the quality of nutritional services in other settings have highlighted poor adherence to guidelines leading to substandard health outcomes [14, 15, 18]. Therefore identifying areas of substandard quality of care is an important step towards improvement of health services [18–23]. In Uganda there is limited published literature on the performance of health facilities offering nutritional services, especially in a refuge setting. The aim of this study was to carry out an assessment of the quality of care provided to children admitted with acute malnutrition at out-patient therapeutic care (OTC) level in Arua district, West Nile region.

## Methods

### Study design, population and setting

This was a cross sectional study and is reported according to the STROBE guidelines. It was conducted between July and August 2016 in Arua district. According to the 2014 census, the estimated population in the district is 808,745 residents, [24]. By May 2017, the district was hosting approximately 174,396 refugees mainly from South Sudan and DR Congo [4]. For this assessment, six health facilities were selected out of the 55 government owned facilities based on the following criteria; those providing nutrition services, those with the highest reported number of malnourished children according to the Health Management and Information System (HMIS) data for the financial year 2016 (July 2015 to June 2016) [25] and whose staff agreed to participate. Exclusion criteria included difficult to access facilities and those without a staff assigned to be responsible for nutrition service delivery.

### Data collection tools, procedures and variables

#### Nutrition service delivery

The Nutrition Service Delivery Assessment (NSDA) was the main tool used for this evaluation. The tool was developed by the Uganda MoH with support from external partners as the official national instrument for assessing performance of nutritional services [26]. It assesses 10 key capacity areas of nutrition service relevant at outpatient level, including: general information on service implementation, adequate human resources, provision of nutritional services, community linkage, quality improvement activities, materials and supplies, nutrition unit requirements, store management, logistics management for commodities, monitoring and evaluation. Data sources include: direct observation, documents review, interviews with health staff, village health teams (VHTs) and mothers of children diagnosed with malnutrition. For each chapter, using strict criteria specified in the tool (similar to checklists), a final judgment on the quality of the services was made and a final score assigned as one of four pre-defined categories: poor, fair, good and excellent. The tool also guides the identification of specific gaps in service delivery in each of the capacity areas.

The study team involved in the NSDA assessment included a senior paediatrician, a nutritionist and a public health expert, all experienced in the National IMAM guidelines [2] and in the use of the NSDA tool [26].

#### Health outcomes

Health outcomes were extracted from the HMIS by a national HMIS focal person for the review period (financial year 2016), according to six categories based on the national definitions in the IMAM guidelines [2]: 1) Cured: attaining a weight-for-height ≥ − 2 standard deviation (SD) from the mean based on the WHO 2006 standards or mid upper circumference (MUAC) of ≥12.5 cm; 2) Non-responders: not reaching discharge criteria after three months or four months for the HIV/TB patients; 3) Defaulters: absent for 2 consecutive follow up visits; 4) Transferred to in-patient care (ITC): condition has deteriorated and requires in-patient care or not responding to treatment; 5) Transferred to to another out-patient care facility (OTC): patient transferred to other nearby OTCs or as requested by caregiver; and 6) Died: patient died while in the program.

#### Quality of case management and quality of data

Quality of case management and quality of data were assessed for each child enrolled in the program during the 2016 financial year using the Integrated Nutrition Register (INR) as a source of data. The INR is the official register at the health facility level where all information on malnourished children is recorded. Data extraction was conducted by a team of six data collectors, trained for this purpose, and directly supervised by a nutritionist. Data collection tools were pre-defined and pilot tested, and standard operating procedures (SOP) were developed to standardise the data extraction process. Quality of case management was assessed using the national guidelines as reference for standards [2] and using five pre-defined process outcomes:1) Correct diagnosis: correct assignment of the category of malnutrition based on weight-for-height Z-score or MUAC as follows: MAM if weight-for-height Z-score > − 3 and < − 2 standard deviation or MUAC (6 to 59 months) > 11.5 and < 12.5 cm and no bilateral pitting oedema; and SAM if weight-for-height Z-score < − 3 Standard deviation or MUAC (6 to 59 months) < 11.5 cm, bilateral pitting oedema, no medical complications and passes appetite test; 2) Correct treatment: correct treatment of cases with SAM such as: 10% glucose/sugar for hypoglycaemia at triage, Amoxicillin for bacterial infections on first day, Measles vaccination on admission (if > 9 months and not yet received), Vitamin A capsule given once at discharge, Iron and folic acid prescribed in presence of anaemia, Mebendazole/Albendazole for helminthic infections on second visit and Ready to Use Therapeutic Foods (RUFT) called Plumpy nut, as main malnutrition prescription; 3) Correct evaluation of HIV: HIV test performed on all patients following the national testing algorithm [27]; 4) Correct counselling of care givers/patients on key messages: delivery of counselling in the following area, as for the national guideline [2]: nutrition, RUFT administration, hygiene, HIV; and 5) Correct exit health outcome assigned: correct assignment of the exit criteria as for the national guideline [2] criteria, as follows: cured, non-respondent, defaulted, transfer to in-patient care or out-patient care and died.

Data quality was assessed using the following two pre-defined indicators: 1) data completeness defined for each single case as “complete” if in information on the following 15 key required fields were filled in: date, patient name, type of nutritional management, nutritional status at enrolment, HIV status at enrolment, anti-retroviral therapy services at enrolment, visit date, oedema, weight, height/length, MUAC colour, Z-score, therapeutic feeds, target exit criteria, exit outcome; and 2) internal consistency defined for each single case as “consistent” if i) the height of the child was consistent over time (ie not decreasing) and ii) the date of the visits was consistent over time (ie progressive dates in the register).

### Data management

Data was collected and double entered into pre-formatted excel spreadsheets and checked for consistency and accuracy by two supervisors before analysis. The distribution of the health facility categorical parameters was presented as frequencies with respective proportions. Health outcomes were assessed against the SPHERE standards [28]. Case management and data quality indicators were assessed against a predefined target of at least 80%. Cases with missing information on health outcomes and quality of case management were counted as incorrect. A two sided *p*-value of < 0.05 was considered as statistically significant.

## Results

### Characteristics of the health facilities

**Fig. 1:**
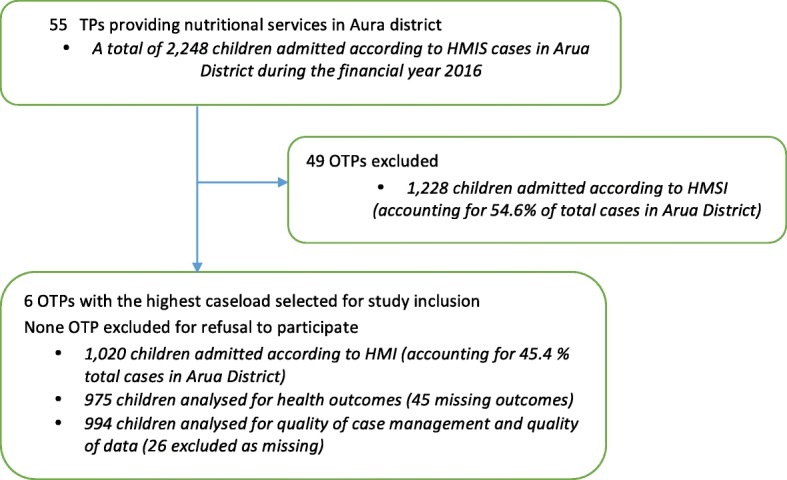
Study flow diagram

The selected population sample, from the six facilities, accounted for 45.4% (1020/2248) of total caseload of malnourished children treated in Arua district during this review period (Fig. 1).

Characteristics of the health facilities are reported in Table 1. Overall, the number of children treated in each facility varied (from 318 to 61) but this was not directly proportional to the estimated population coverage (number of children diagnosed per 1000 population coverage ranging from 2.8 to 32.8).

**Table 1:** Key characteristics of the health facilities

Variable	Health facility						
	HC 1	HC 2	HC 3	HC 4	HC 5	HC 6	Totals
Health Center level^a^	IV	III	III	III	III	III	–
Estimated population coverage	32,000	3960	22,548	13,779	2500	21,662	96,449
Children diagnosed with acute malnutrition^b^	318	292	116	151	82	61	1020
Number of staff assigned to the nutritional unit	2	2	3	1	3	2	13
Nutritional staff qualification							
Clinical officer	1	0	0	0	0	0	1
Enrolled nurse/midwife	1	1	1	0	3	2	8
Nursing assistant	0	1	2	1	0	0	4
Staff trained in IMAM guideline	1	0	0	0	0	0	1

^a^Levels of primary health care in Uganda is tiered into health center I,II,III and IV

^b^HMIS data July 2015 – June 2016 (financial year)

Four out of six facilities had two or less staff assigned to the nutrition unit, with only one facility having a clinical officer involved. Overall, only one staff (7.6%) had been trained in the IMAM guidelines.

### Nutrition service delivery assessment

**Fig. 2:**
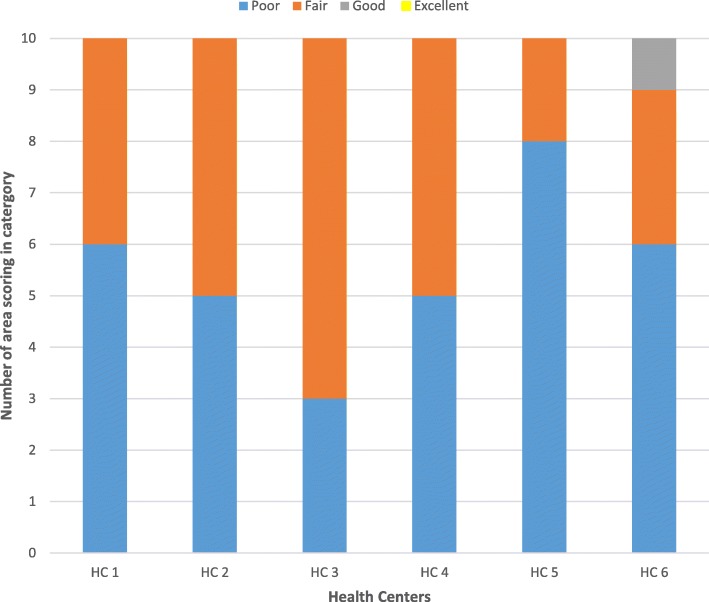
Distribution of NSDA scores by facility

All facilities except two scored either poor or fair under all the 10 assessment areas of the NSAD tool (Table 2). Overall, 33/60 (55.0%) areas were scored as poor, 25/60 (41.7%) as fair, 2/60 (3.3%) as good, and none as excellent. In particular, the following two areas were scored as poor in all facilities: quality improvement activities and monitoring and evaluation (see Additional file 1). Figure 2 shows a summary the distribution of the NSDA scores.

**Table 2:** Performance of health facilities in the selected capacity areas

Capacity area	Health facility Score^a^					
	HC 1	HC 2	HC 3	HC 4	HC 5	HC 6
1. General information on service implementation	Fair	Good	Fair	Fair	Fair	Fair
2. Adequate human resources	Poor	Poor	Poor	Poor	Fair	Poor
3. Provision of nutritional services	Fair	Fair	Fair	Poor	Fair	Poor
4. Community Linkagetable	Fair	Fair	Fair	Poor	Poor	Good
5. Quality improvement activities	Poor	Poor	Poor	Poor	Poor	Poor
6. Materials and Supplies	Poor	Fair	Poor	Poor	Fair	Poor
7. Nutrition unit requirements	Fair	Fair	Poor	Fair	Fair	Poor
8. Store management	Poor	Fair	Fair	Poor	Fair	Fair
9. Logistics Management for commodities	Poor	Poor	Fair	Poor	Fair	Poor
10. Monitoring and evaluation	Poor	Poor	Poor	Poor	Poor	Poor

^a^Score performance categories according to the NSDA tool: poor; fair; good; excellent [26]

Table 3 shows the specific gaps in quality of nutritional services identified with the NSDA. Key findings included: poor organisation of services at the nutrition service delivery point; poor case diagnosis and treatment; stock out of nutrition foods; weak community linkage mechanisms.

**Table 3:** Gaps in quality of nutritional services observed by capacity area

Capacity area	Observed issue
Organisation of services	Nutrition services delivered “under the tree”
	Working hours unclear
	Frequent service delays if rain
	No triage
	Chaotic organisation, no clear roles and responsibilities
	No transport for children sent In-patient Therapeutic Care (ITC)
	Working hours unclear
Case management	Triage not performed
	Mid Upper Aram Circumference (MUAC) not routinely done at all entry points (Out patients department -OPD, Tuberculosis and Anti retroviral therapy - TB/ART)
	Mis-classification SAM/MAM
	Z-score never used (only MUAC used)
	No history taking
	Comprehensive clinical examination as per the Integrated Management of Childhood Illnesses (IMCI) not performed
Treatment	Water with sugar not offered at admission
	10 key messages on RUTF not delivered
	Individual counselling never performed
	Amoxicillin, vitamine A, Iron and mebendazole not prescribed
	MAM and SAM usually treated the same
Integrated Management of childhood Illnesses (IMCI)	HIV status often indicated as unknown despite availability of testing kits
	TB rarely assessed
	Children at OPD not always assessed for nutritional status
	Children with malnutrition not assessed according to IMCI
	Staff working in out-patient care not trained in IMCI
	Old IMCI job aids in some facilities
Supplies	Stock out of RUTF observed in many facilities
	Lack of mean of transport to facilities
	Lack of timely request from facilities
Staffing	Lack of staffing with some facilities having no nutritional focal person appointed
	Lack of nutritional specific training
	Poor practices even among trained staff
	Village Health Teams (VHTs) usually not formally trained but doing the job at OTC in place of facility staff
Community linkage	VHTs screening reports not readily available
	Blank VHTs registers
	No effective means of communication between facilities and village health teams (VHTs)
	No incentives for the VHT
Quality improvement	Several supportive supervision activities are conducted on a quarterly basis, at facilities but only few are specific to nutrition

Abbreviations: *ART* Anti Retro-viral Therapy, *HIV* Human Immune-deficiency Virus, *IMCI* Integrated Management of Childhood Illness, *ITC* In-patient Therapeutic Care, *MAM* Moderate Acute Malnutrition, *MUAC* Mid-Upper Arm Circumference, *OPD* Out Patients Department, *OTC* Out-patient Therapeutic Care, *RUTF* Ready-to-Use Therapeutic Foods, *SAM* Severe Acute Malnutrition, *TB* Tuberculosis, *VHT* Village Health Teams

Of note, the assessment also identified some areas with good service delivery. All health facilities were using HMIS forms (INR and monthly quarterly reports), had basic nutrition equipment (weighing, length/height measuring scales and MUAC tapes), essential job aids (Z-score classification and counselling aids), VHTs were engaged and there was evidence of quarterly supervision conducted by district health team.

### Health outcomes

**Fig. 3:**
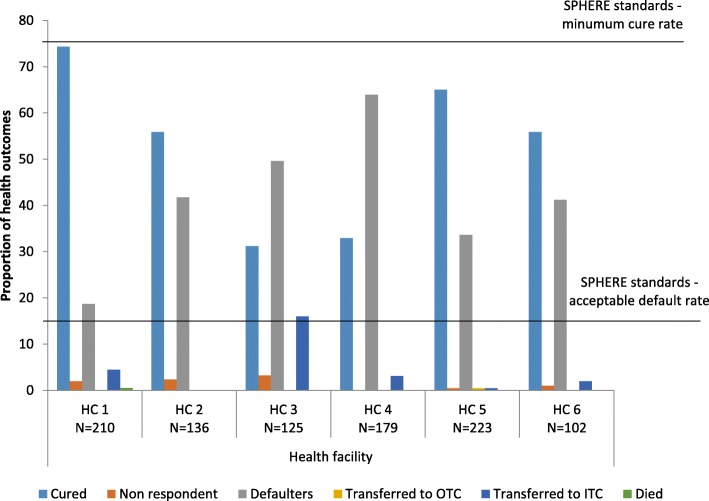
Distribution of health outcomes

The distribution of health outcomes is shown in Fig. 3. The cure rate and defaulter rate were the two health outcomes that were predominantly assigned (see Additional file 1). The overall cured rate in all the six health facilities was 52.9% while the overall defaulting rate was 38.2%. Significant heterogeneity was observed between these outcomes across health centers with the cure rate ranging from 31.2 to 74.4% and the defaulting rate ranging from 18.7 to 63.9%. During the entire study period, 37 children (4.0%) were transferred to ITC, 13 (1.3%) were classified as non-responders and only one participant (0.1%) was recorded to have died.

### Quality of case management

Overall, 994 cases of malnourished children were identified in the INR and reviewed (see Additional file 1). Health facility performance on all case management process indicators was highly heterogeneous across facilities (Table 4). The rate of correct diagnosis ranged from 4.5 to 91.2%, correct treatment from 0 to 50.0%, correct HIV status assignment from 58.1 to 91.2%, correct counselling from 11.2 to 99.3% and correct exit outcome from 0 to 75.9%. The overall average rates were as follows: correct diagnosis at 34.6%; correct treatment at 19.2%; correct counselling at 47.6%; correct evaluation of HIV at 73.5% and correct exit outcome at 16.7%. None of the overall estimates for process outcomes reached the pre-defined target of 80% with a statistically significant difference when compared to this threshold (chi *p*-value = 0.001).

**Table 4:** Case management and data quality

Variable	Health facility							
	HC 1 *N* = 194	HC 2 *N* = 137	HC 3^a^ No data	HC 4 *N* = 301	HC 5 *N* = 228	HC 6 *N* = 134	Total *N* = 994	Chi *p*-value^#^
Correct process outcomes								
Diagnosis	30(15.5)	125(91.2)	–	88(29.2)	95(41.7)	6(4.5)	344(34.6)	0.001
IMAM treatment	0	0	–	151(50.0)	28(12.3)	12(9.0)	191(19.2)	0.001
Evaluation of HIV	158(81.4)	90(65.7)	–	175(58.1)	208(91.2)	100(74.6)	731(73.5)	0.001
Counselling of patients	39(20.1)	136(99.3)	–	172(57.1)	111(48.7)	15(11.2)	473(47.6)	0.001
Exit outcome assigned	19(9.8)	104(75.9)	–	31(10.3)	12(5.3)	0	166(16.7)	0.001
Data quality								
Data completeness	0	44(32.1)	–	0(0)	0(0)	0(0)	44(4.4)	0.001
Data consistency	0	120(87.6)	–	74(25.6)	11(4.8)	1(0.8)	206(20.7)	0.001

Data source: Integrated Nutrition Register

^a^Data not available in the integrated nutrition register

#*p*-value assessed against a pre-defined target of 80% achievement

### Data quality

There was high heterogeneity across health centers in data quality. Data completeness ranged from 0 to 32.1% and data consistency ranged from 0 to 87.6% (Table 4). The overall mean completeness rate was 4.4% and consistency at 20.7% with both indicators far below the pre-defined threshold of 80% (chi *p*-value = 0.001).

### Additional analysis

No clear correlation could be found between single indicators (NSDA scores, cured rate, process outcomes, quality of data) and the type of health center (level IV vs III), or the volume of work (number of children admitted). No clear internal correlation among different indicators could be found (performance of the different indicators did not seem to be directly linked to each other).

## Discussion

This is the first study reporting on the performance of nutritional services for children in Arua district. The assessment shows that, even though some positive aspects were observed, there are substantial deficiencies in the quality of nutrition services at health center level in Arua district. Significant gaps were observed both by using the national tool for Nutrition Service Delivery Assessment (NSDA) and by reviewing key indicators of health outcomes, case management and data quality in the official records.

The observed health facility cure rate of 52.9% was far below the international SPHERE standards target set at above 75% [28], while the defaulting rate of 38.3%, was significantly higher than the standard’s target set at below 15% (28). One of the possible reasons for this low cure rate may be the lack of adherence to guidelines for case management as observed in this study. Important clinical practices such as triage, screening of all children for malnutrition, history taking, detailed examination, diagnosis of SAM and MAM, individual counselling, complementary treatment and assignment of exit outcomes were not being performed according to the IMAM guidelines [2]. Additionally, laboratory screening for HIV and TB was not routinely conducted, despite the availability of laboratory diagnostic kits. Such poor performance of quality of health service delivery has also been reported in other studies both in routine settings in Uganda [13, 15, 29, 30] and in refugee settings such as in Ethiopia [31–34].

Another key reason explaining the poor performance of case management, in addition to inadequate human resource, is the substantial lack of training of heath facility staff, both frequently observed challenges in low and middle income countries [35]. The impact of targeted training on both health workers performance and children outcomes is relatively well documented. For example, a systematic review examining the effectiveness of nutritional training of health workers showed a clear benefit in improving feeding frequency, energy intake, and dietary diversity of children [36].

Notably, almost all the assessed health facilities had basic nutritional equipment such as digital weighing scales, length/height measuring boards, MUAC tapes and essential job aids. However, the frequent stock out of RUTF, an essential nutrition management commodity, was a significant issue, a finding in line with two earlier studies conducted in other regions in Uganda [29, 30].

The observed challenges such as stock out of RUTF, poor organisation of services including irregular working hours and long waiting times and weak community linkages re-affirm some of the underlying factors explaining the very high defaulting rate observed [29]. The poor performance of VHTs especially regarding case-identification and referral of cases is an observation that deserves further scrutiny because this study was not designed to identify the causes of this occurrence. However, evidence from a systemic review on factors that influence performance of community health workers (CHWs) such as VHTs found that lack of supervision, lack of training and lack of financial incentives were the main barriers to achieving an acceptable performance from CHWs [37]. Minimizing such barriers would improve access to care and ultimately the achievement of better health outcomes. Evidence shows that barriers to access for service users may increase mortality, especially among children with SAM who actually requires urgent medical attention [38].

Poor data quality is another important but frequently reported problem in low income countries, including Uganda [39, 40]. Good quality data is the basis for evidence based decision making and two suggested approaches for improvement in such settings include better training on data quality assurance procedures and intensive supportive supervision [38–41].

As already documented, the influx of refugees into a community negatively affects the performance of health services in such settings [31, 32]. However it is also true that poor performance has been reported in settings experiencing no refugee crisis [29, 30], indicating that refugee circumstances is not the sole explanation for such a performance. This study did not aim at comparing the performance of nutritional service before and during the most recent refugee crisis In Arua, but rather at collecting baseline data for service delivery evaluation. Future studies should aim at monitoring health system performance over time while exploring the influence of different factors on key outcomes.

Limitations of this study included the relatively small sample size in terms of health facilities, however, the study sample population captured over 45% of cases of children admitted to nutritional services in Arua district. Even though most of the assessment was conducted by direct evaluation using the NSDA tool [26], health outcomes and case management were assessed using recorded data, which, by nature, are exposed to a risk of recall bias. We tried to minimised this bias in different ways such as choosing the official documents as data sources with the expectation that all information of each child with malnutrition was recorded, using trained data collectors, using pre-defined data collection variables, developing standard operating procedures and transparency during reporting of study findings.

Recommendations for policy makers derived from this study may include: hiring and training of health facility staff to fill the human resource gap; strengthening supportive supervision to improve performance at different levels (case management, timely requests of RUTF, data quality, community linkages); and conducting regular NSDA assessments to monitor progress over time. More studies are needed to identifying effective approaches to enhance adherence to national guidelines and ultimately improve health outcomes of children.

## Conclusion

This assessment revealed that quality of care and health outcomes of children with malnutrition in Arua district are far below the internationally acceptable SPHERE standards. Significant deficiencies were observed under organization of service, case management, procurement, community linkage and data quality. In the future both researchers and policy makers should aim at identifying effective approaches to increase quality of care for children with malnutrition in Arua district and similar settings.

## Additional file

Additional file 1: Quantitative dataset. (XLSX 57 kb)

## Abbreviations

ART: Anti Retroviral Therapy
DHT: District Health Team
HC: Health Center
HIV: Human Immuno-defiency Virus
HMIS: Health Management and Information System
IMAM: Integrated Management of Acute Malnutrition
IMCI: Integrated Management of Childhood Illness
INR: Integrated Nutrition Register
ITC: In-patient Therapeutic Care
MAM: Moderate Acute Malnutrition
MoH: Ministry of Health
MUAC: Mid Upper Arm Circumference
NSDA: Nutrition Service Delivery Assessment
OPD: Out Patient Department
OTC: Out-patient Therapeutic Care
RUTF: Reay to Use Therapeutic Foods
SAM: Sever Acute Malnutrition
TB: Tuberculosis
UNICEF: United Nations International Emergency Fund
VHT: Village Health Team
WHO: World Health Organisation
